# Supplementary material for: Overemphasis on recovery inhibits community transformation and creates resilience traps
Source: Nat Commun. 2021 Dec 17;12:7331. doi: 10.1038/s41467-021-27359-5 (PMC8683504; doi:10.1038/s41467-021-27359-5)
Supplement: Supplementary file 1 — Supplementary Information [file 41467_2021_27359_MOESM1_ESM.pdf]

Supplement for Manuscript *Overemphasis on recovery inhibits community transformation and creates resilience traps*

Resilience Calculation Figures

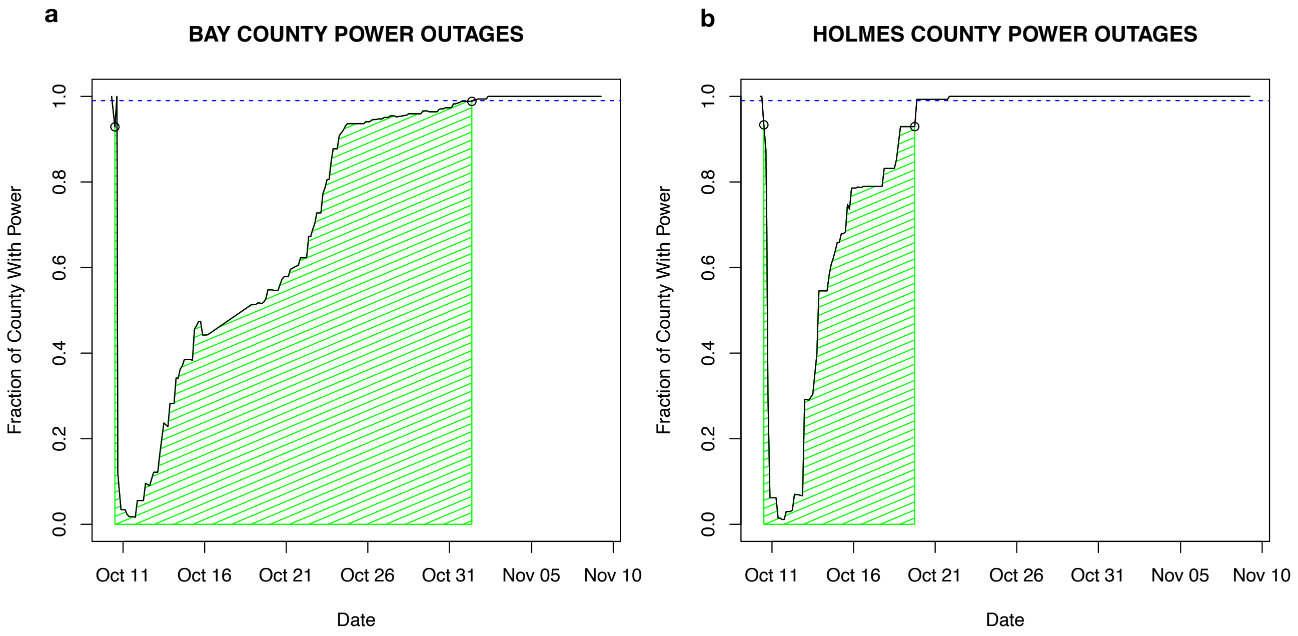

Supplementary Figure 1. Power Outages During Hurricane Michael for Bay (a) and Holmes (b) County, FL

Examples of the resilience calculation performed for each county. For each county, the value $Q(t)$ is the fraction of the county with access to power at time t; represented in these figures as the solid black lines. The time of initial disruption, t_0 is the first time in which $Q(t)$ drops below 0.99 (a pre-defined threshold; represented in Supplemental Figure 1a and 1b as the left-most black circle. Similarly, t_f is the first post-disruption time in which $Q(t)$ exceeds 0.99 and is shown in the above figures as the right-most black circles. The numerator of the Eq. 1 for calculating R_{county}, $\int_{t_0}^{t_f} Q(t)$, is shaded green in Supplemental Figs. 1a and 1b. $t_f - t_0|$, the denominator of R_{county}, is the duration of time between the initial disruption and repair time.

Model selection plots and comparisons

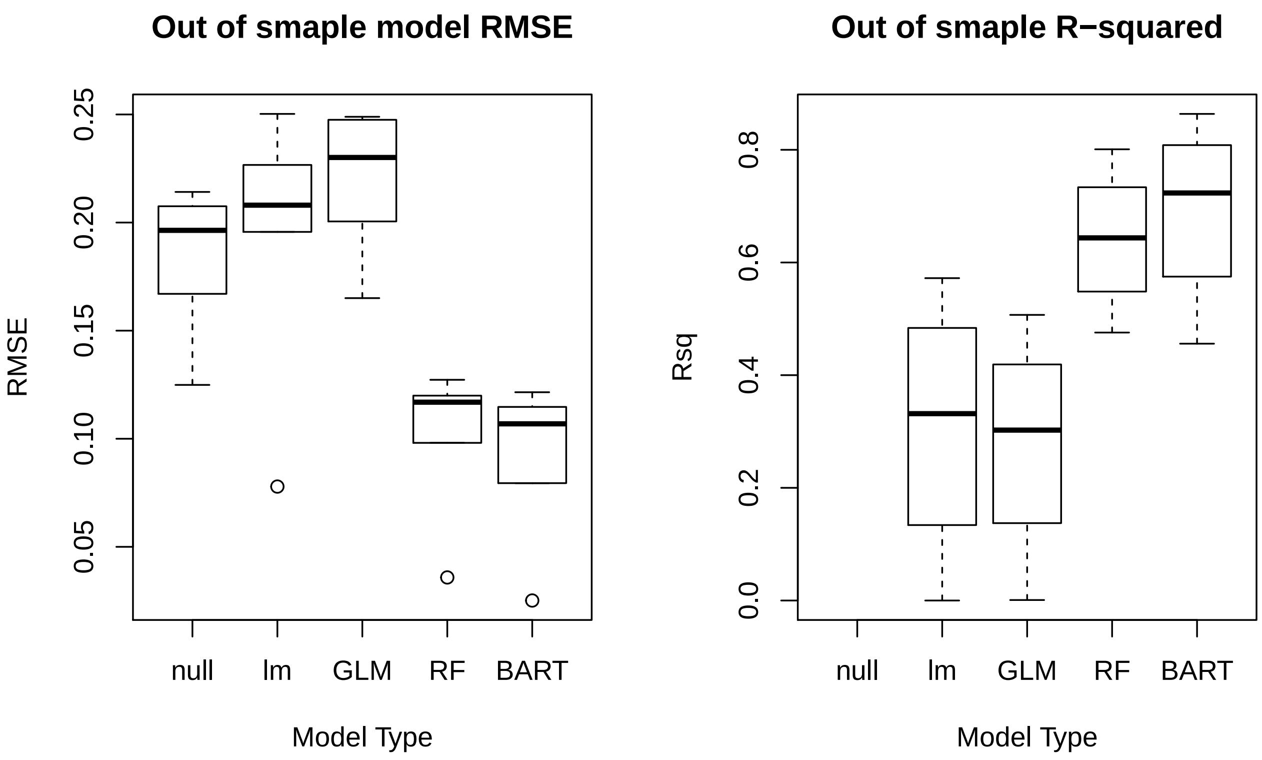

Supplementary Figure 2. Out of sample RMSE and R^2 for the prediction of engineering resilience as a function of the selected community risk factors. Note the null model's out of sample R^2 is 0 because the standard deviation of predictions is 0. Boxes represent 25th to 75th percentiles (the IQR), horizontal black lines are means, whiskers show 1.5 times the IQR, and points are outlying.

Community Risk Factors

Supplementary Table 1. Original Input Variables

Category	Variable Name	Description	Year (s)	Source
Demographics	Population	County level population	2018	American Community Survey, Table: B01003[1]
	Racial Inequity	Kolm's Inequality Measure of racial demographcs of census tracts in each county	2018	American Community Survey,Table: B02001[1]
	Citizenship	County level fraction of population who are citizens	2018	American Community Survey, Table: B05001[1]
Housing	1-Year Housing Tenure	County level fraction of population living in the same home for more than 1 year	2018	American Community Survey, Table: B07001[1]
	Within County Relocation	County level fraction of population who moved within the state in the past year	2018	American Community Survey, Table: B07001[1]
	Within State Relocation	County level fraction of population who moved from outside the county but within the state in the past year	2018	American Community Survey, Table: B07001[1]
	Out of State Relocation	County level fraction of population who moved from outside the state but within the US in the past year	2018	American Community Survey, Table: B07001[1]
	Abroad Relocation	County level fraction of population who moved from outside the US in the past year	2018	American Community Survey, Table: B07001[1]
	Household Size, Renters	County level average household size of renter-occupied housing units	2018	American Community Survey, Table: B25010[1]
	Household Size, Owners	County level average household size of owner-occupied housing units	2018	American Community Survey, Table: B25010[1]
	Rents Dwelling	County level fraction of population in renter-occupied housing	2018	American Community Survey, Table: B25008[1]
Mobility	Commuting Alone	County level fraction of population who primarily commutes in a car, truck or van alone	2018	American Community Survey, Table: B08101[1]
	Carpool Commute	County level fraction of population who primarily commutes by carpooling in a car, truck, or van	2018	American Community Survey, Table: B08101[1]
	Public Transportation Commuting	County level fraction of population who primarily commutes via public transportation (excluding taxis)	2018	American Community Survey, Table: B08101[1]
	Walking Commute	County level fraction of population who primarily commutes by walking	2018	American Community Survey, Table: B08101[1]

	Bike, cab, other commuting	County level fraction of population who primarily commutes by taxicab, motorcycle, bicycle, or other means	2018	American Community Survey, Table: B08101[1]
	Working From Home	County level fraction of population who primarily works from home	2018	American Community Survey, Table: B08101[1]
Educational Attainment	High School Degree	County level fraction of population over 25 with a regular high school diploma	2018	American Community Survey, Table: B15003[1]
	GED	County level fraction of population over 25 with a GED or alternative credential	2018	American Community Survey, Table: B15003[1]
	Associates Degree	County level fraction of population over 25 with an Associate's degree	2018	American Community Survey, Table: B15003[1]
	Bachelor's Degree	County level fraction of population over 25 with a Bachelor's degree	2018	American Community Survey, Table: B15003[1]
Language	Speaks English	County level fraction of population 5 years and older speaking only English at home	2018	American Community Survey, Table: B16007[1]
	Speaks Spanish	County level fraction of population 5 years and older speaking Spanish at home	2018	American Community Survey, Table: B16007[1]
	Speaks Indo-European	County level fraction of population 5 years and older speaking other Indo-European Languages at home	2018	American Community Survey, Table: B16007[1]
	Speaks API	County level fraction of population 5 years and older speaking Asian and Pacific Island Languages at home	2018	American Community Survey, Table: B16007[1]
Income	Income Inequality	County level Gini Index of income inequality	2018	American Community Survey, Table: B19083[1]
	Aggregate Household Income	County level aggregate household income in the past 12 months in 2018 inflation-adjusted dollars	2018	American Community Survey, Table: B19025[1]
	Income Deficit	County level income deficit	2018	American Community Survey, Table: B17008[1]
	Income Through Earning	County level fraction of households with income from wage or salary income	2018	American Community Survey, Table: B19051[1]
	Income Through Interest	County level fraction of households with income through interest, dividends or net rental income	2018	American Community Survey, Table: B19054[1]
	Income Through SSI	County level fraction of households with Social Security income	2018	American Community Survey, Table: B19054[1]

Communication	Internet Access	County level fraction of households with internet access	2018	American Community Survey, Table: B28002[1]
	Cellphone Only	County level fraction of households with only a cellular data plan with no other type of internet subscription	2018	American Community Survey, Table: B28002[1]
	No Internet Access	County level fraction of households without internet access	2018	American Community Survey, Table:B28002[1]
Climate Change Opinions	Climate Opinions	County level PCA decomposition of positive responses to climate change-related polls	2019	Yale Program on Climate Change Communication[2]

In addition, the census-tract level data was collected for each ACS variable and Kolm's inequality measure[3] was computed for each county and variable. 1-year Data from the American Community Survey are collected from January 1st 2018 to December 31st 2018 for populations of 20,000 or more.

Supplementary Table 2. Resilience Values

Florida County	Engineering Resilience
ALACHUA	0.9259779
BAKER	1.0000000
BAY	0.6339356
BRADFORD	0.8244461
BREVARD	0.9860843
BROWARD	1.0000000
CALHOUN	0.4019630
CHARLOTTE	1.0000000
CITRUS	1.0000000
CLAY	1.0000000
COLLIER	1.0000000
COLUMBIA	0.9881542
DESOTO	1.0000000
DIXIE	1.0000000
DUVAL	1.0000000
ESCAMBIA	0.9681062
FLAGLER	1.0000000
FRANKLIN	0.3629637
GADSDEN	0.5167954
GILCHRIST	0.4537804
GLADES	1.0000000
GULF	0.5326955
HAMILTON	0.7993632
HARDEE	1.0000000
HENDRY	1.0000000
HERNANDO	1.0000000
HIGHLANDS	1.0000000
HILLSBOROUGH	1.0000000
HOLMES	0.5542315
INDIAN	0.8273789
JACKSON	0.4582192
JEFFERSON	0.5209264
LAFAYETTE	0.8433915
LAKE	1.0000000
LEE	1.0000000
LEON	0.6739230
LEVY	1.0000000
LIBERTY	0.4777898
MADISON	0.8937640
MANATEE	1.0000000
MARION	1.0000000
MARTIN	1.0000000
MIAMI-DADE	1.0000000
MONROE	1.0000000
NASSAU	1.0000000
OKALOOSA	0.9813354
OKEECHOBEE	1.0000000
ORANGE	1.0000000
OSCEOLA	1.0000000
PALM	1.0000000
PASCO	1.0000000
PINELLAS	1.0000000
POLK	1.0000000
PUTNAM	1.0000000
SANTA	1.0000000
SARASOTA	1.0000000
SEMINOLE	1.0000000
ST JOHNS	1.0000000
ST LUCIE	1.0000000
SUMTER	1.0000000
SUWANNEE	0.9570933
TAYLOR	0.8497418
UNION	0.9877392
VOLUSIA	1.0000000
WAKULLA	0.6641285
WALTON	0.8435221
WASHINGTON	0.7034305

Example Temporal Trajectory

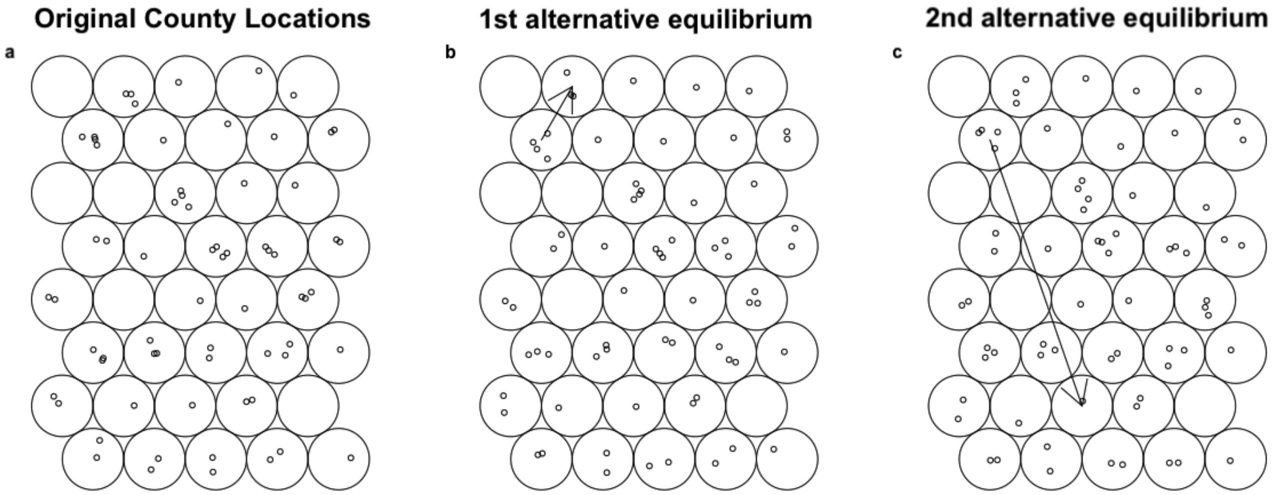

Supplementary Figure 3. Example County Movement. (a) shows the location of nodes with no perturbation, (b) shows the temporal trajectory of Bay County associated with the shift to the lowest alternative- equilibrium seen in Figure**??** c. (c) is the temporal trajectory for the change to the highest alternative-equilibrium seen in Figure **??**c. The length of the arrows in (b) and (c) are the magnitudes of transformation for each perturbation.

CCN Node Size

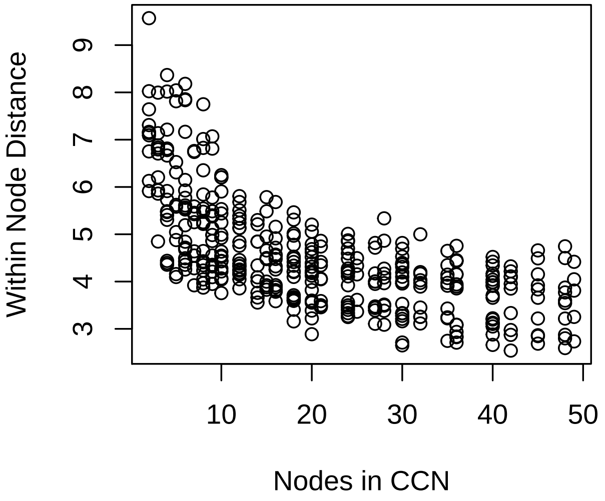

Supplementary Figure 4. Within-node distance as a function of sample size. The y-axis shows the mean distance between each point and its nearest node, while the x-axis shows the number of nodes used to train the CCN.

References

1. Bureau, U. C. American Community Survey 5-year estimates. Accessed June 23rd, 2020.
2. Howe, P. D., Mildenberger, M., Marlon, J. R. & Leiserowitz, A. Geographic variation in opinions on climate change at state and local scales in the USA. **5**, 596–603, DOI: 10.1038/nclimate2583.
3. Zeileis, A. ineq: Measuring Inequality, Concentration, and Poverty (2014). R package version 0.2-13.
